# Comparative Effects of Low-Level Laser Therapy and Transcutaneous Electrical Nerve Stimulation on Neurosensory Recovery After Trigeminal Nerve Injury: An Exploratory Non-Randomized Clinical Study

**DOI:** 10.3390/jcm15031049

**Published:** 2026-01-28

**Authors:** Mert Zeytinoğlu, Alpay Savran, Burhanettin Uludag

**Affiliations:** 1 Department of Oral Surgery, Faculty of Dentistry, Ege University, 35040 Izmir, Turkey; 2 Oral and Maxillofacial Surgery Clinic, 61100 Trabzon, Turkey; alpaysavran@yahoo.com; 3 Department of Neurology, Faculty of Medicine, Ege University, 35100 Izmir, Turkey; burhanettin.uludag@ege.edu.tr

**Keywords:** inferior alveolar nerve, lingual nerve, third molar surgery, low-level laser therapy, transcutaneous electrical nerve stimulation

## Abstract

**Objective:** Inferior alveolar (IAN) and lingual nerve (LN) injuries are known complications of impacted mandibular third molar surgery and may result in persistent neurosensory deficits. This exploratory, non-randomized clinical study evaluated the clinical and electrophysiological effects of low-level laser therapy (LLLT) and transcutaneous electrical nerve stimulation (TENS) on neurosensory recovery following trigeminal nerve injury. **Methods:** Twenty-seven patients with postoperative IAN or LN injury received LLLT, TENS, or placebo therapy according to institutional clinical protocols. Clinical outcomes were assessed using Visual Analog Scale (VAS) scores, and electrophysiological evaluation was performed using electromyography by measuring cutaneous silent period (CSP) duration. Non-parametric statistical analyses were conducted using the Wilcoxon signed-rank and Mann–Whitney U tests. **Results:** LLLT was associated with statistically significant improvements in several neurosensory symptoms, including pain, burning sensation, speech difficulty, biting, and taste disturbance. In contrast, TENS and placebo treatment did not demonstrate a consistent or generalized improvement across neurosensory outcomes. CSP durations differed significantly between healthy and pathological sides both before and after treatment. Although CSP duration showed a tendency to increase following LLLT, these changes did not reach statistical significance. Subgroup analysis revealed greater clinical improvement in LN injuries compared with IAN injuries within the LLLT group. **Conclusions:** Within the limitations of this exploratory study, LLLT was associated with more pronounced clinical improvement than TENS or placebo in patients with third molar-related trigeminal nerve injury. CSP measurements provided supportive objective information, although electrophysiological recovery remained limited.

## 1. Introduction

Surgical procedures involving the mandibular region—particularly impacted third molar extractions—are among the most frequently performed interventions in oral and maxillofacial surgery. Despite advances in surgical planning and technique, injury to the inferior alveolar nerve (IAN) and lingual nerve (LN) remains a well-recognized complication of dentoalveolar surgery [1,2]. The incidence of postoperative neurosensory disturbances varies widely depending on surgical difficulty, anatomical proximity of the nerve, and assessment methodology, yet even transient nerve injury may adversely affect daily functioning [3,4].

Damage to the IAN or LN may result in a range of sensory abnormalities, including hypoesthesia, paresthesia, dysesthesia, and neuropathic pain, often accompanied by functional impairments such as speech disturbances, impaired mastication, accidental biting, and altered taste [5,6]. Peripheral nerve injury initiates complex pathophysiological processes involving axonal degeneration, demyelination, neuroinflammation, and altered afferent excitability. While mild injuries may resolve spontaneously, recovery is often prolonged or incomplete in cases of axonotmesis or partial neurotmesis, leading to persistent positive sensory symptoms and reduced quality of life [7,8].

Accurate evaluation of trigeminal nerve dysfunction is essential for determining injury severity and guiding management. Beyond clinical neurosensory testing, electrophysiological methods provide objective insight into sensory–motor interactions. In particular, electromyographic assessment of trigeminal reflexes, including analysis of the cutaneous silent period, reflects afferent pathway integrity and inhibitory mechanisms and has been shown to be sensitive to neuropathic involvement following mandibular nerve injury [9,10].

Given the limitations of spontaneous recovery and the invasive nature of microsurgical repair, interest has increasingly focused on non-invasive therapeutic modalities aimed at enhancing nerve regeneration and symptom control. Transcutaneous electrical nerve stimulation (TENS) is widely used for pain modulation through segmental and suprasegmental inhibitory pathways and has demonstrated efficacy in various chronic pain conditions; however, evidence supporting its benefit in peripheral trigeminal nerve injury remains limited and inconsistent [11,12].

Low-level laser therapy (LLLT), also known as photobiomodulation, has emerged as a promising adjunctive treatment for peripheral nerve injury. Experimental and clinical studies indicate that LLLT may enhance axonal regeneration, modulate inflammatory responses, and improve mitochondrial function and neural conduction without thermal tissue damage [13,14,15]. In maxillofacial surgery, several reports have described improved neurosensory recovery following IAN or LN injury with LLLT, although treatment parameters and outcome measures vary considerably [16,17,18].

Despite increasing interest in these modalities, direct comparative data evaluating the electrophysiological and clinical effects of TENS versus LLLT on trigeminal nerve recovery remain scarce. Therefore, the present study aims to compare the effects of TENS and low-level laser therapy on neurosensory recovery following inferior alveolar and lingual nerve injury using combined clinical and electrophysiological assessments, thereby contributing to a more evidence-based approach to non-invasive management of trigeminal nerve injuries.

Based on the available evidence, the working hypothesis of this study was that low-level laser therapy (LLLT) would result in greater clinical improvement in neurosensory symptoms compared with transcutaneous electrical nerve stimulation (TENS) or placebo following inferior alveolar and lingual nerve injury. The null hypothesis was that there would be no significant differences among treatment modalities in clinical or electrophysiological outcomes.

## 2. Materials and Methods

### 2.1. Study Design and Patient Population

This study was conducted as a prospective, non-randomized clinical investigation. All interventions were performed in accordance with the treatment protocols of the Department of Physical Therapy and Rehabilitation, Faculty of Medicine, Ege University. The study was approved by the Institutional Review Board of Ege University Faculty of Dentistry (code: 193372; date of approval 25 April 2006). A total of 27 patients who developed neurosensory disturbances of the inferior alveolar nerve (IAN) or lingual nerve (LN) following surgical removal of impacted mandibular third molars were included in the study (Table 1).

All patients were referred to the clinic due to persistent postoperative neurosensory symptoms and had no systemic disease. Prior to inclusion, all patients were informed about the electrophysiological assessments and physical therapy interventions, and written informed consent was obtained.

**Figure 1:**
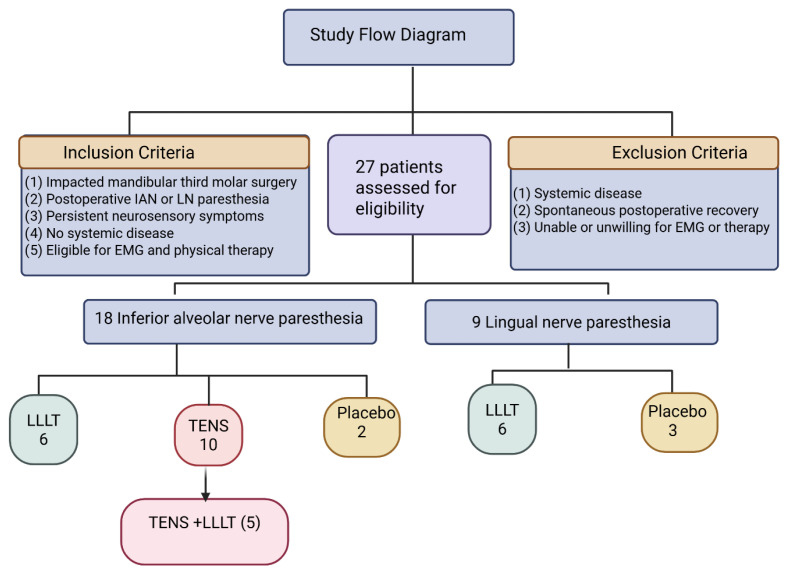
Study flow diagram of patient enrollment and treatment allocation. This diagram illustrates the patient eligibility assessment, inclusion and exclusion criteria, classification by nerve involvement, and treatment allocation according to the institutional clinical protocol. A total of 27 patients were assessed for eligibility. Patients were grouped according to inferior alveolar or lingual nerve paresthesia and received low-level laser therapy (LLLT), transcutaneous electrical nerve stimulation (TENS), placebo treatment, or adjunctive laser therapy following TENS, as indicated. Patients who received adjunctive laser therapy after TENS were analyzed as a separate subgroup.

Patients with systemic diseases, neurological disorders, or a history of peripheral neuropathy were excluded. All participants were referred for evaluation and treatment during the postoperative period due to persistent sensory complaints (Table 1, Figure 1).

#### 2.1.1. Inclusion Criteria

Patients were eligible for inclusion if they met all of the following criteria:

History of impacted mandibular third molar surgery.

Presence of inferior alveolar nerve or lingual nerve paresthesia in the postoperative period.

Persistence of neurosensory symptoms without spontaneous recovery during postoperative follow-up.

Absence of systemic disease.

Ability and willingness to undergo electrophysiological evaluation (EMG) and physical therapy interventions (LLLT and/or TENS).

#### 2.1.2. Exclusion Criteria

Patients were excluded from the study if they met any of the following criteria:

Presence of systemic disease.

Spontaneous postoperative recovery of neurosensory symptoms.

Inability or refusal to undergo electrophysiological assessment or physical therapy treatment.

#### 2.1.3. Distribution According to Nerve Involvement

Inferior alveolar nerve (lower lip paresthesia): 18 patients.

Lingual nerve (tongue paresthesia): 9 patients.

#### 2.1.4. Treatment Allocation

All patients underwent baseline electrophysiological evaluation prior to the initiation of physical therapy. This study was conducted as a pragmatic, clinically driven treatment allocation rather than a randomized controlled trial.

Based on clinical presentation, 18 patients presented with lower lip paresthesia related to inferior alveolar nerve injury, and 9 patients presented with tongue paresthesia related to lingual nerve injury.

Among patients with lip paresthesia, 6 received low-level laser therapy (LLLT), 10 received transcutaneous electrical nerve stimulation (TENS), and 2 received placebo treatment. Among patients with tongue paresthesia, 6 received LLLT and 3 received placebo treatment.

In addition, five patients who initially received TENS and demonstrated insufficient clinical improvement subsequently underwent adjunctive laser therapy during the treatment course. Patients who received adjunctive laser therapy following TENS were analyzed as a separate subgroup (TENS + LLLT), in accordance with the original clinical protocol.

#### 2.1.5. Control (Placebo) Group

The control group consisted of 5 patients (2 with inferior alveolar nerve involvement and 3 with lingual nerve involvement). These patients underwent routine clinical and electrophysiological follow-up but did not receive any active physical therapy intervention. In the placebo group, devices were applied without active laser emission or electrical stimulation.

### 2.2. Therapeutic Interventions and Electrophysiological Evaluation

#### 2.2.1. Electrophysiological Evaluation (EMG)

Electromyographic (EMG) recordings were performed at the EMG Laboratory of the Neurophysiology Department, Faculty of Medicine, Ege University, following standard neurophysiological protocols. A four-channel Nicolet Viking-IV EMG system was used with 10 mm diameter Ag/AgCl (silver chloride) cup electrodes.

Recordings were obtained using a monopolar technique, with the active electrode positioned over the muscle belly and the reference electrode placed on an electrically neutral area. Stimulation was delivered using a surface bipolar stimulator with a 2 cm interelectrode distance at the supraorbital foramen, mental foramen, and intraoral trigonum retromolare. Recordings were obtained bilaterally from the ipsilateral and contralateral orbicularis oculi muscles.

Both painful and painless stimuli were applied. Painful stimuli primarily activated A-delta and C fibers, whereas painless stimuli activated A-beta fibers, reflecting trigeminal sensory reflex activity. This electrophysiological protocol represents a reflex activity recording, in which reproducible muscle responses were obtained following sensory nerve stimulation.

**Figure 2:**
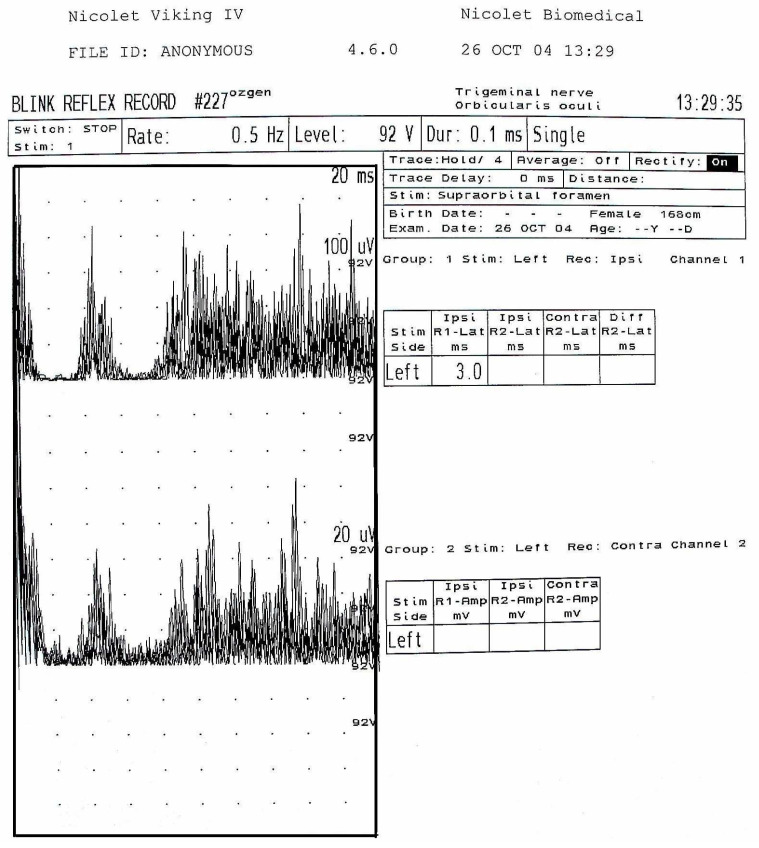
Devices and electrophysiological assessment used in the study. Representative blink reflex electromyography (EMG) recording obtained using a Nicolet Viking IV system, showing ipsilateral and contralateral orbicularis oculi muscle responses following supraorbital nerve stimulation.

Signals were recorded in raw form and subsequently rectified by mirroring negative potentials, as commonly applied in reflex studies. Recordings were obtained with a sweep time of 200 ms and a sensitivity of 100 mV/division, adjusted to 100 ms and 50 mV/division when required (Figure 2).

The cutaneous silent period (CSP) represents an inhibitory reflex response following sensory nerve stimulation. In the present study, total silent period durations (SP1 + SP2) were measured and compared between the healthy and pathological sides.

#### 2.2.2. Low-Level Laser Therapy (LLLT)

Low-level laser therapy was administered using a Gallium–Aluminum–Arsenide (GaAlAs) diode laser system (Endolaser 476^®^, Enraf-Nonius, Rotterdam, The Netherlands) in accordance with the clinical protocol routinely applied at the Department of Physical Therapy and Rehabilitation during the study period. GaAlAs diode lasers operate within the near-infrared spectrum and are widely utilized in photobiomodulation applications targeting peripheral nerve recovery.

Laser irradiation was delivered at a wavelength of 830 nm in continuous wave mode (100%), as continuous emission ensures stable power delivery, whereas pulsed operation with reduced duty cycles may decrease average output power. The device provided a continuous output power of 100 mW. Each irradiation point was treated for 40 s, corresponding to a total energy delivery of 4 J per point. Considering the probe’s active irradiation area (approximately 0.4–1.0 cm^2^), the resulting energy density fell within the commonly accepted therapeutic range of 4–10 J/cm^2^ per application point.

Laser application was performed using a contact technique, with the probe positioned perpendicular to the skin or mucosal surface over the affected nerve distribution areas. In patients with inferior alveolar nerve involvement, irradiation sites included the mental foramen region and the mandibular body along the anatomical course of the nerve. For lingual nerve involvement, laser therapy was applied intraorally to the lingual mucosa adjacent to the mandibular third molar region.

Laser therapy was administered at three predefined anatomical points along the injured nerve pathway. Treatment sessions were conducted five times per week over a four-week period, yielding a total of 20 sessions. To specifically target the neurosensory recovery phase, laser therapy was initiated after the resolution of postoperative edema and trismus. Standard laser safety precautions were observed throughout all sessions, including the use of protective eyewear by both patients and operators [19].

#### 2.2.3. Transcutaneous Electrical Nerve Stimulation (TENS)

Transcutaneous electrical nerve stimulation (TENS) therapy was administered in accordance with the clinical protocol applied at the Department of Physical Therapy and Rehabilitation during the study period. TENS was delivered using high-frequency stimulation with a frequency range of 50–100 Hz, a short pulse duration of 40–75 μs, and a low-intensity current of 10–30 mA, adjusted individually to patient tolerance. Stimulation was delivered using a symmetrical biphasic rectangular waveform.

**Figure 3:**
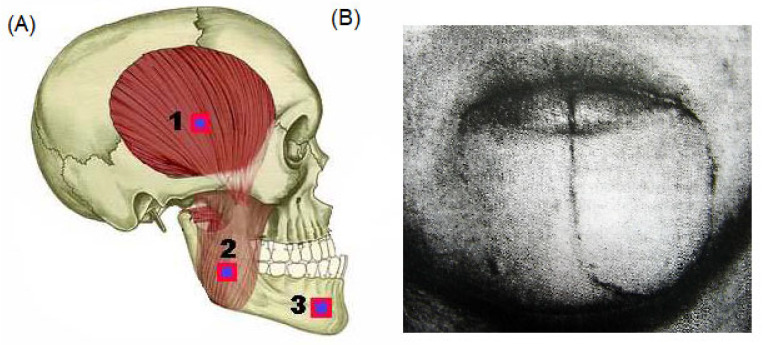
Extraoral application sites used for TENS and low-level laser therapy. (**A**) Schematic illustration showing the extraoral transcutaneous electrical nerve stimulation (TENS) electrode placement sites over the masseter muscle and mandibular region corresponding to the anatomical course of the inferior alveolar nerve. (**B**) Representative image demonstrating the extraoral low-level laser therapy (LLLT) application sites for inferior alveolar nerve involvement.

Stimulation was applied extraorally on the side of nerve injury using bipolar carbon–silicone alloy electrodes, with a conductive gel placed between the electrodes and the skin to facilitate current transmission. Electrodes were positioned over the masseter muscle and mandibular region, corresponding to the anatomical course of the affected trigeminal nerve branches, as illustrated in Figure 3A.

TENS was applied at two predefined extraoral points, with each point stimulated for approximately 5 min. Treatment sessions were administered five times per week, with a total treatment duration of four weeks (20 sessions).

Throughout all sessions, patients were monitored for comfort and tolerability, and stimulation intensity was maintained at a level producing a strong but comfortable sensory perception without inducing muscle contraction.

TENS parameters were kept constant throughout the treatment period for each patient. Electrode placement, stimulation frequency, pulse duration, and session duration were standardized according to institutional clinical protocols and were not modified during follow-up.

### 2.3. Post-Treatment Patient Follow-Up and Evaluation

After completion of the treatments for each patient group in accordance with the physical therapy protocols defined above, criteria such as pain, spontaneous recovery, conduction disturbances, and functional impairment were evaluated using electromyographic tests and Visual Analog Scale (VAS) scores. Post-treatment evaluations were performed immediately after completion of the 4-week therapy protocol.

### 2.4. Total VAS Score Evaluation

Neurosensory symptoms were assessed using the Visual Analog Scale (VAS), with each symptom rated from 0 (no symptom) to 10 (maximum severity). For each patient, a total VAS score (SUM) was calculated by summing the VAS scores of all assessed neurosensory symptoms, providing a composite measure of overall symptom burden. Patient-level total VAS (SUM) scores were used for statistical analysis. The primary outcome of the study was the change in total VAS score from baseline to post-treatment. Pre-treatment and post-treatment total VAS (SUM) scores were compared within each treatment group using the Wilcoxon signed-rank test.

### 2.5. Statistical Analysis

Statistical analyses were performed using SPSS software (version 19; IBM Corp., Armonk, NY, USA). Data distribution was evaluated by visual inspection and the Shapiro–Wilk test. Given the small sample size and the predominance of non-normally distributed variables, non-parametric statistical methods were applied throughout the analysis.

Within-group pre- and post-treatment comparisons of neurosensory symptom scores and cutaneous silent period (CSP) durations were conducted using the Wilcoxon signed-rank test. Between-group comparisons (LLLT vs. TENS) and subgroup analyses according to nerve type (inferior alveolar nerve [IAN] vs. lingual nerve [LN]) were performed using the Mann–Whitney U test. Paired analyses were restricted to measurements obtained from the same anatomical side (healthy or pathological).

Neurosensory outcomes were assessed using Visual Analog Scale (VAS) scores for individual symptoms. In addition, a composite total VAS score, calculated by summing all symptom-specific VAS ratings, was predefined as the primary clinical outcome, reflecting the overall neurosensory symptom burden. Individual symptom analyses were considered secondary and exploratory.

Electrophysiological assessment included measurement of total cutaneous silent period duration (SP1 + SP2) using electromyography (EMG). CSP durations were compared between healthy and pathological sides and across time points to assess changes in trigeminal inhibitory reflex function.

To address the issue of multiple statistical comparisons arising from the assessment of numerous neurosensory symptoms, a Holm–Bonferroni correction was applied to secondary outcome analyses involving individual symptom VAS scores. This method was selected as it provides control of family-wise error while preserving greater statistical power than the classical Bonferroni adjustment in studies with correlated outcomes and limited sample sizes. Adjusted *p*-values are reported where applicable. Analyses of the predefined primary outcome (total VAS score) and electrophysiological CSP parameters were not subjected to multiplicity correction.

All statistical tests were two-tailed. A corrected *p* value < 0.05 was considered statistically significant.

## 3. Result

### 3.1. Patient Characteristics and Group Distribution

A total of 27 patients with postoperative neurosensory deficits were included (18 IAN, 9 LN). Among IAN cases, 6 received LLLT, 10 received TENS (including 5 later treated with adjunct LLLT), and 2 received placebo. Among LN cases, 6 received LLLT and 3 received placebo. Patient allocation is summarized in the study flow diagram (Figure 1).

### 3.2. Clinical Outcomes After Low-Level Laser Therapy (LLLT)

Statistical analyses of the patient group treated with laser therapy were performed based on the parameters presented in Table 2 and Table 3, comparing pre-treatment and post-treatment outcomes. In the LLLT group, no statistically significant pre–post differences were observed for itching, tickling sensation, salivation, vibration, warm sensation, and cold sensation according to Wilcoxon signed-rank test results (Table 2).

In contrast, statistically significant differences were identified for numbness, pain, stabbing sensation, electric shock sensation, tension, stiffness, crawling sensation, tingling, burning sensation, speech difficulty, accidental biting, and taste disturbance when comparing pre-treatment and post-treatment assessments (Table 3).

### 3.3. Clinical Outcomes After Transcutaneous Electrical Nerve Stimulation (TENS)

The pre-treatment and post-treatment evaluations for the patient group treated with TENS therapy are presented in Table 4 and Table 5. Among the 10 patients who received TENS therapy, statistically significant differences were observed in numbness, tension, crawling sensation, cold sensation, tingling, burning sensation, speech difficulty, and taste disturbance according to the Wilcoxon signed-rank test results (Table 5).

### 3.4. Clinical Outcomes in Patients Receiving Laser Therapy After TENS

The pre-treatment and post-treatment statistical analyses for the patient group that received laser therapy after TENS treatment are shown in Table 6. According to the Wilcoxon signed-rank test results, significant differences were found post-treatment in parameters such as stiffness, tingling, speech difficulty, and accidental biting. On the other hand, no changes were observed post-treatment for numbness, pain, stabbing sensation, electric shock, tension, crawling sensation, cold sensation, warm sensation, itching, tickling, burning sensation, vibration, and salivation and no significant differences were found in the Wilcoxon signed-rank test.

### 3.5. Between-Group Comparisons: LLLT Versus TENS

**Figure 4:**
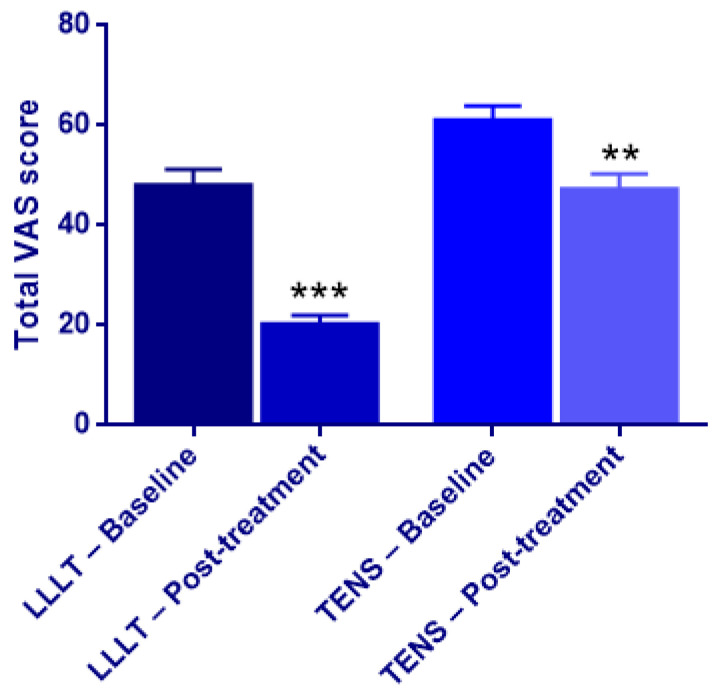
Bar graph showing baseline and post-treatment total VAS scores in the LLLT and TENS groups. Data are presented as mean ± standard deviation. ** *p* < 0.01, *** *p* < 0.001 vs. baseline (Wilcoxon signed-rank test).

The Mann–Whitney U test analyses comparing the laser-treated and TENS-treated groups are summarized in Table 7 and Table 8. Although statistically significant between-group differences were observed for tension and salivation at baseline, these isolated findings did not indicate a systematic or clinically meaningful difference between the laser and TENS groups across the overall neurosensory profile prior to treatment (Table 7, Figure 4).

In contrast, in the laser-treated group, post-treatment reductions were observed in the mean, median, and standard deviation values of symptom scores. Mann–Whitney test analyses demonstrated statistically significant improvements in tension, stiffness, crawling sensation, cold sensation, speech difficulty, salivation, and accidental biting following laser therapy, whereas no significant differences were detected between pre- and post-treatment values for the remaining symptoms (Table 8, Figure 4).

### 3.6. Subgroup Analysis According to Nerve Type (IAN vs. LN)

#### 3.6.1. Lingualis vs. Alveolaris Inferior (Laser-Treated Patients)

**Figure 5:**
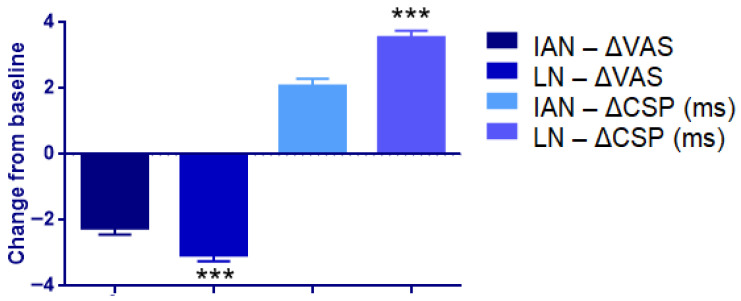
Subgroup analysis according to nerve type in the LLLT group. Bar graph illustrating changes from baseline in VAS scores (ΔVAS) and CSP durations (ΔCSP) among patients with inferior alveolar nerve (IAN) and lingual nerve (LN) injuries. Negative ΔVAS values denote symptom reduction, whereas positive ΔCSP values indicate electrophysiological recovery. *** *p* < 0.001 between nerve subgroups (Mann–Whitney U test).

Table 9 presents the comparison of neurosensory symptoms between patients with N. lingualis and N. alveolaris inferior injury following laser therapy. According to the Mann–Whitney U test results, statistically significant differences were observed for biting, crawling sensation, speech difficulty, and taste disturbance, with higher symptom severity noted in patients with N. lingualis involvement (*p* < 0.05). In contrast, no statistically significant differences were detected between the two nerve injury groups for numbness, pain, electric shock sensation, tension, stiffness, cold sensation, warm sensation, itching, tingling, swallowing, burning sensation, vibration, or salivation (Figure 5).

#### 3.6.2. N. Lingualis: Pre- vs. Post-Laser Therapy

Changes in neurosensory symptoms before and after laser therapy in patients with N. lingualis injury are summarized in Table 10. Wilcoxon signed-rank test analysis demonstrated statistically significant post-treatment improvements in biting, speech difficulty, and taste disturbance (*p* < 0.05). Although reductions in median values were also observed for pain, electric shock sensation, tension, stiffness, crawling sensation, tingling, and salivation, these changes did not reach statistical significance.

#### 3.6.3. N. Alveolaris Inferior: Pre- vs. Post-Laser Therapy

Table 11 shows the pre-treatment and post-treatment neurosensory outcomes in patients with N. alveolaris inferior injury who received laser therapy. According to the Wilcoxon signed-rank test results, statistically significant reductions were observed for biting, crawling sensation, and speech difficulty (*p* < 0.05). Although decreases in median values were also noted for electric shock sensation, tension, stiffness, tingling, and salivation, these changes did not reach statistical significance.

#### 3.6.4. Post-Laser Comparison: N. Lingualis vs. N. Alveolaris Inferior

Post-treatment comparisons between N. lingualis and N. alveolaris inferior groups following laser therapy are presented in Table 12. Mann–Whitney U test results revealed statistically significant differences in biting, speech difficulty, and taste disturbance, with more pronounced residual symptoms observed in the N. lingualis group (*p* < 0.05). No significant between-group differences were found for numbness, pain, electric shock sensation, tension, stiffness, crawling sensation, tingling, or salivation.

#### 3.6.5. Laser-Placebo Group—Wilcoxon Signed-Rank Test (Table 9, Table 10, Table 11 and Table 12)

In the patient group that received placebo laser treatment, Wilcoxon signed-rank test analyses revealed no statistically significant differences between pre-treatment and post-treatment assessments for any neurosensory parameter (Table 9, Table 10, Table 11 and Table 12).

#### 3.6.6. Cutaneous Silent Period (CSP) Analyses

**Figure 6:**
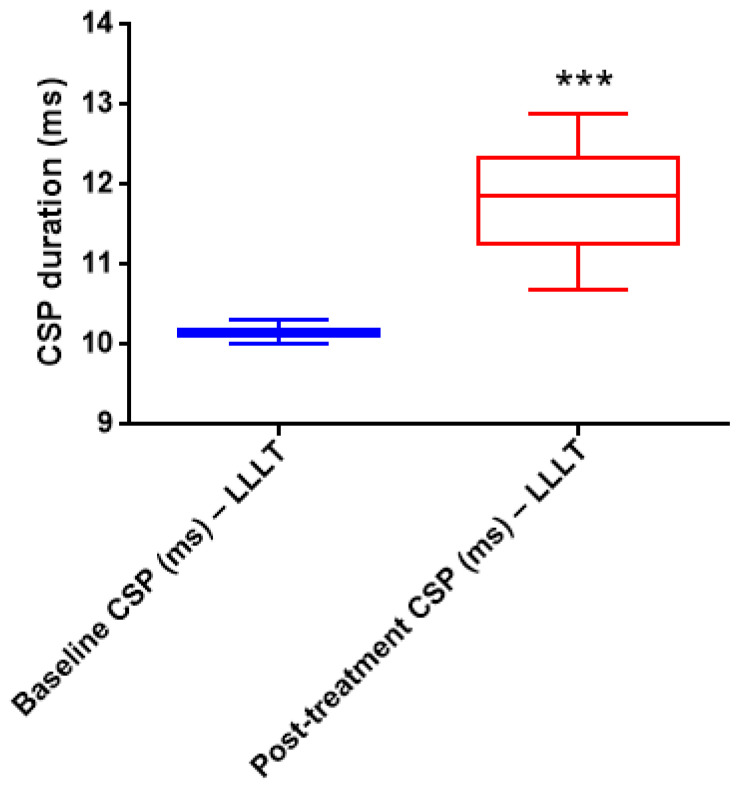
Cutaneous silent period (CSP) duration on the pathological side. Box-and-whisker plot showing CSP duration before and after treatment in the LLLT group. Boxes represent the median and interquartile range, and whiskers indicate minimum and maximum values. *** *p* < 0.001 vs. baseline (Wilcoxon signed-rank test).

Wilcoxon signed-rank test demonstrated significantly longer EMG cutaneous silent period (CSP) durations on the healthy side compared with the pathological side for both the masseter and mylohyoid muscles across all painful tasks and most painless tasks before treatment (*p* < 0.05). The only exception was the painless repetitive movement task of the mylohyoid muscle, in which no statistically significant difference was observed (*p* = 0.066) (Table 13) (Figure 6).

When pre- and post-treatment CSP durations were compared on the pathological side, no statistically significant changes were detected for the masseter muscle across any task (*p* > 0.05). In contrast, the mylohyoid muscle showed statistically significant prolongation of CSP durations during painful soft bite, painful maximal force, and painless maximal force tasks following treatment (*p* < 0.05) (Table 14).

## 4. Discussion

The present exploratory study evaluated the clinical and electrophysiological effects of low-level laser therapy (LLLT) and transcutaneous electrical nerve stimulation (TENS) in patients with inferior alveolar and lingual nerve injuries following third molar surgery. The findings indicate that LLLT was associated with greater improvement in neurosensory symptoms, as reflected by reductions in VAS scores, whereas TENS and placebo did not demonstrate a consistent or generalized clinical benefit.

The observed clinical improvement following LLLT is consistent with established mechanisms of photobiomodulation, including enhanced mitochondrial ATP production, modulation of inflammatory pathways, and support of axonal repair without thermal damage [13,16,20,21]. Previous clinical studies have similarly reported improved neurosensory outcomes after LLLT in trigeminal nerve injuries, although treatment parameters and outcome measures vary across studies [16,20].

Not all investigations have reported a beneficial effect of LLLT. Miloro et al. observed no significant difference between laser and placebo treatment following inferior alveolar or lingual nerve injury [17]. Differences in laser parameters, treatment timing, and study design may partly explain these discrepancies, underscoring the need for standardized protocols and adequately powered trials.

The divergent outcomes observed between LLLT and TENS in the present study can be explained by their fundamentally different therapeutic targets. LLLT primarily acts through biomodulatory and regenerative mechanisms, including enhancement of mitochondrial activity, attenuation of neuroinflammation, and support of axonal repair. In contrast, TENS exerts its effects mainly via neuromodulatory pain-gating pathways that transiently alter afferent signal transmission without directly promoting structural nerve recovery. This mechanistic distinction provides a biologically plausible explanation for the more sustained neurosensory improvement observed following LLLT compared with the limited effects of TENS. Within the broader context of post-procedural neural complication management, LLLT aligns with emerging mechanism-driven, minimally invasive strategies, including recent biomaterial-based approaches such as Jasminum-derived nano-reinforced systems designed to modulate postoperative pain and inflammation [22].

Consistent with this mechanistic distinction, TENS did not show a consistent or clinically meaningful effect on overall neurosensory recovery in the present cohort. Although TENS is effective for pain modulation in various craniofacial conditions, its primary mechanism involves sensory gating rather than direct promotion of nerve regeneration, which may limit its utility in chronic trigeminal nerve injury [23,24].

An additional observation was the greater clinical improvement observed in lingual nerve injuries compared with inferior alveolar nerve injuries within the LLLT group. This finding is in line with previous reports and may relate to anatomical or biological differences between these nerves; however, given the small subgroup sizes, this result should be interpreted cautiously.

Electrophysiological evaluation using cutaneous silent period (CSP) measurements was included to provide objective information on trigeminal sensory inhibitory pathways. CSP and related electromyographic assessments are primarily used to localize nerve dysfunction, assess inhibitory reflex integrity, and aid in prognostication in peripheral nerve disorders rather than to directly demonstrate treatment-related recovery [25]. In the present study, CSP durations differed between healthy and pathological sides; however, within-group pre- and post-treatment changes did not reach statistical significance in most comparisons. Therefore, these electrophysiological findings should be interpreted as exploratory in nature and do not provide statistically significant evidence of treatment efficacy. While CSP measurements offered complementary physiological context, they cannot be considered confirmatory of the observed clinical improvements following LLLT.

Taken together, the results suggest that LLLT may offer a clinically relevant non-invasive option for neurosensory symptom management following trigeminal nerve injury, whereas TENS appears to have limited impact under the applied parameters. Larger randomized studies with standardized intervention protocols and longer follow-up are required to clarify these findings.

### Limitations

This study has several limitations. It was conducted as an exploratory, non-randomized clinical investigation with treatment allocation based on clinical availability, resulting in small and uneven group sizes and limited baseline comparability without statistical adjustment. In addition, some patients initially treated with TENS subsequently received adjunct low-level laser therapy, introducing crossover exposure and restricting clear comparisons between LLLT, TENS, and placebo. Follow-up evaluations were performed at a single and relatively short post-treatment time point; therefore, delayed clinical or electrophysiological recovery, particularly in cutaneous silent period (CSP) parameters, may not have been captured. Neurosensory outcomes were assessed using VAS-based symptom ratings combined into a composite score, which was not derived from a validated neurosensory instrument and may be susceptible to expectation bias in the absence of blinding. Moreover, CSP assessment was exploratory and reflected inhibitory reflex integrity rather than direct treatment-induced recovery. Finally, the small sample size limited statistical power, especially for subgroup analyses. Larger randomized studies with standardized protocols, validated outcomes, longer follow-up, and appropriate blinding are required.

## 5. Conclusions

In this exploratory clinical study, low-level laser therapy (LLLT) was associated with more pronounced and consistent improvement in neurosensory symptoms compared with transcutaneous electrical nerve stimulation (TENS) or placebo following third molar-related trigeminal nerve injury. Clinical benefits of LLLT were evident across multiple VAS-based outcomes, whereas TENS showed limited and inconsistent effects. Electrophysiological cutaneous silent period measurements provided supportive objective information but did not demonstrate statistically significant recovery. These findings suggest that LLLT may represent a clinically useful non-invasive option for neurosensory symptom management. Larger, randomized studies with standardized protocols and longer follow-up are required to confirm these results and define optimal treatment parameters.

## Figures and Tables

**Table 1:** Baseline Characteristics of the Study Population.

Variable	Value
Number of patients	27
Sex (male/female)	7/20
Age range (years)	25–45
Nerve injury type	IAN: 18, LN: 9
Time since injury	≥1 month
Etiology	Impacted mandibular third molar surgery

**Table 2:** Descriptive statistics of neurosensory symptoms before and after laser therapy.

Symptom	Time Point	Mean ± SD	Median	Min–Max
Numbness	Pre-treatment	8.17 ± 0.72	8.00	7–9
	Post-treatment	5.92 ± 1.56	6.00	4–8
Pain	Pre-treatment	1.75 ± 2.05	1.00	0–5
	Post-treatment	0.42 ± 0.67	0.00	0–2
Stabbing sensation	Pre-treatment	3.67 ± 3.11	3.50	0–8
	Post-treatment	1.75 ± 2.26	0.50	0–7
Electric shock sensation	Pre-treatment	2.58 ± 2.19	3.00	0–7
	Post-treatment	0.92 ± 1.44	0.50	0–5
Tension	Pre-treatment	2.33 ± 2.96	0.00	0–7
	Post-treatment	0.67 ± 0.99	0.00	0–3
Stiffness	Pre-treatment	4.00 ± 3.10	5.00	0–7
	Post-treatment	1.33 ± 1.44	1.00	0–4
Crawling sensation	Pre-treatment	2.00 ± 2.63	0.00	0–7
	Post-treatment	0.50 ± 0.91	0.00	0–3
Cold sensation	Pre-treatment	0.25 ± 0.87	0.00	0–3
	Post-treatment	0.00 ± 0.00	0.00	0–0
Warm sensation	Pre-treatment	1.50 ± 2.32	0.00	0–6
	Post-treatment	0.75 ± 1.49	0.00	0–5
Itching	Pre-treatment	0.75 ± 1.77	0.00	0–5
	Post-treatment	0.42 ± 1.17	0.00	0–4
Tingling	Pre-treatment	6.08 ± 1.17	6.00	3–7
	Post-treatment	2.50 ± 1.62	2.00	1–7
Tickling	Pre-treatment	0.25 ± 0.87	0.00	0–3
	Post-treatment	0.25 ± 0.87	0.00	0–3
Burning sensation	Pre-treatment	2.08 ± 2.31	1.50	0–6
	Post-treatment	0.75 ± 1.36	0.00	0–4
Speech difficulty	Pre-treatment	4.83 ± 1.40	4.50	3–7
	Post-treatment	2.00 ± 1.54	2.00	0–5
Salivation	Pre-treatment	0.75 ± 1.42	0.00	0–4
	Post-treatment	0.08 ± 0.29	0.00	0–1
Accidental biting	Pre-treatment	4.25 ± 1.91	4.00	0–7
	Post-treatment	1.08 ± 1.08	1.00	0–3
Taste disturbance	Pre-treatment	2.92 ± 3.09	2.50	0–7
	Post-treatment	1.00 ± 1.13	0.50	0–3

Data are presented as mean ± standard deviation (SD), median, and range (minimum–maximum). Pre- and post-treatment comparisons were performed using the Wilcoxon signed-rank test.

**Table 3:** Wilcoxon Signed-Rank Test Results According to VAS Criteria.

Variable	Asymp. Sig. (2-Tailed)	Exact Sig. (2-Tailed)	Exact Sig. (1-Tailed)
Numbness-1 Numbness-2	0.003	0.001	0.000
Pain-1 Pain-2	0.026	0.031	0.016
Stabbing sensation-1 Stabbing sensation-2	0.016	0.016	0.008
Electric shock sensation-1 Electric shock sensation-2	0.010	0.008	0.004
Tension-1 Tension-2	0.039	0.063	0.031
Stiffness-1 Stiffness-2	0.011	0.008	0.004
Crawling sensation-1 Crawling sensation-2	0.034	0.063	0.031
Cold sensation-1 Cold sensation-2	0.317	1.000	0.500
Warm sensation-1 Warm sensation-2	0.144	0.250	0.125
Itching-1 Itching-2	0.180	0.500	0.250
Tingling-1 Tingling-2	0.003	0.001	0.001
Tickling-1 Tickling-2	1.000	1.000	1.000
Burning sensation-1 Burning sensation-2	0.034	0.063	0.031
Vibration-1 Vibration-2	1.000	1.000	1.000
Speech difficulty-1 Speech difficulty-2	0.004	0.002	0.001
Salivation-1 Salivation-2	0.102	0.250	0.125
Accidental biting-2	0.003	0.001	0.000
Taste disturbance-1 Taste disturbance-2	0.024	0.031	0.016

Wilcoxon signed-rank test results for VAS-based neurosensory criteria in the LLLT-treated group.

**Table 4:** Descriptive statistics of neurosensory symptoms before and after TENS therapy.

Symptom	Time Point	Mean ± SD	Median	Min–Max
Numbness	Pre-treatment	8.10 ± 0.74	8.00	7–9
	Post-treatment	6.90 ± 2.03	7.00	2–9
Pain	Pre-treatment	1.90 ± 3.21	0.00	0–8
	Post-treatment	1.70 ± 2.83	0.00	0–7
Stabbing sensation	Pre-treatment	2.10 ± 2.85	0.00	0–7
	Post-treatment	1.80 ± 2.57	0.00	0–7
Electric shock sensation	Pre-treatment	2.50 ± 3.34	0.00	0–8
	Post-treatment	2.10 ± 2.73	0.00	0–6
Tension	Pre-treatment	5.30 ± 2.41	6.00	0–8
	Post-treatment	4.00 ± 2.26	5.00	0–7
Stiffness	Pre-treatment	6.00 ± 1.41	6.50	3–7
	Post-treatment	5.10 ± 2.13	6.00	1–7
Crawling sensation	Pre-treatment	4.60 ± 2.95	5.00	0–8
	Post-treatment	3.10 ± 2.33	3.00	0–7
Cold sensation	Pre-treatment	2.70 ± 3.13	1.50	0–8
	Post-treatment	1.50 ± 1.90	0.50	0–5
Warm sensation	Pre-treatment	0.80 ± 2.53	0.00	0–8
	Post-treatment	0.70 ± 2.21	0.00	0–7
Itching	Pre-treatment	2.10 ± 2.85	0.00	0–7
	Post-treatment	1.40 ± 1.90	0.00	0–5
Tingling	Pre-treatment	4.30 ± 2.63	4.50	0–8
	Post-treatment	3.70 ± 2.31	4.00	0–7
Tickling	Pre-treatment	0.00 ± 0.00	0.00	0–0
	Post-treatment	0.00 ± 0.00	0.00	0–0
Burning sensation	Pre-treatment	1.30 ± 2.83	0.00	0–8
	Post-treatment	1.10 ± 2.42	0.00	0–7
Vibration	Pre-treatment	0.30 ± 0.95	0.00	0–3
	Post-treatment	0.10 ± 0.32	0.00	0–1
Speech difficulty	Pre-treatment	4.90 ± 1.52	5.00	3–7
	Post-treatment	3.70 ± 1.64	4.00	1–6
Salivation	Pre-treatment	3.00 ± 2.36	3.00	0–6
	Post-treatment	2.00 ± 1.76	2.00	0–4
Accidental biting	Pre-treatment	5.60 ± 1.51	5.00	4–8
	Post-treatment	4.20 ± 1.81	4.00	1–6
Taste disturbance	Pre-treatment	5.60 ± 1.51	5.00	4–8
	Post-treatment	4.20 ± 1.81	4.00	1–6

Data are presented as mean ± standard deviation (SD), median, and range (minimum–maximum). Pre- and post-treatment comparisons were performed using the Wilcoxon signed-rank test.

**Table 5:** Wilcoxon signed-rank test results according to VAS criteria (TENS group).

Variable (1–2)	Asymp. Sig. (2-Tailed)	Exact Sig. (2-Tailed)	Exact Sig. (1-Tailed)
Numbness 1	0.024 *	0.031	0.016
Pain (1–2)	0.157	0.500	0.250
Stabbing sensation (1–2)	0.000 *	0.250	0.125
Electric shock sensation (1–2)	0.180	0.500	0.250
Tension (1–2)	0.026 *	0.031	0.016
Stiffness (1–2)	0.109	0.250	0.125
Crawling sensation (1–2)	0.016 *	0.016	0.008
Cold sensation (1–2)	0.042 *	0.063	0.031
Warm sensation (1–2)	0.317	1.000	0.500
Itching (1–2)	0.059	0.125	0.063
Tingling (1–2)	0.014 *	0.031	0.016
Tickling (1–2)	1.000	1.000	1.000
Burning sensation (1–2)	0.157	0.500	0.250
Vibration (1–2)	0.317	1.000	0.500
Speech difficulty (1–2)	0.010 *	0.008	0.004
Salivation (1–2)	0.066	0.125	0.063
Accidental biting (1–2)	0.066	0.125	0.063
Taste disturbance (1–2)	0.024 *	0.031	0.016

Wilcoxon signed-rank test results for VAS-based neurosensory criteria in the TENS-treated group (1–2 denote pre-treatment and post-treatment assessments, respectively). In contrast, no statistically significant differences were detected for pain, stabbing sensation, electric shock sensation, stiffness, warm sensation, itching, tickling, vibration, salivation, or accidental biting when comparing pre-treatment and post-treatment measurements (Table 5). * *p* < 0.05 was considered statistically significant.

**Table 6:** Wilcoxon signed-rank test results according to VAS criteria (TENS followed by laser group).

Variable (1–2)	Asymp. Sig. (2-Tailed)	Exact Sig. (2-Tailed)	Exact Sig. (1-Tailed)
Numbness (1–2)	0.102	0.250	0.125
Pain (1–2)	0.102	0.250	0.125
Stabbing sensation (1–2)	0.180	0.500	0.250
Electric shock sensation (1–2)	0.102	0.250	0.125
Tension (1–2)	0.059	0.125	0.063
Stiffness (1–2)	0.038 *	0.063	0.031
Crawling sensation (1–2)	0.066	0.125	0.063
Cold sensation (1–2)	1.000	1.000	1.000
Warm sensation (1–2)	0.317	1.000	0.500
Itching (1–2)	0.102	0.250	0.125
Tingling (1–2)	0.042 *	0.063	0.031
Tickling (1–2)	1.000	1.000	0.500
Burning sensation (1–2)	0.180	0.500	0.250
Vibration (1–2)	1.000	1.000	1.000
Speech difficulty (1–2)	0.039 *	0.063	0.031
Salivation (1–2)	0.046 *	0.125	0.063
Accidental biting (1–2)	0.041 *	0.063	0.031

Wilcoxon signed-rank test results according to VAS criteria in patients who received laser therapy following TENS treatment (1–2 denote pre-treatment and post-treatment assessments, respectively). * *p* < 0.05 was considered statistically significant.

**Table 7:** Comparison of laser and TENS treatment groups before treatment.

Variable	Mann–Whitney U	Wilcoxon W	Z	Asymp. Sig. (2-Tailed)	Exact Sig. (1-Tailed)
Numbness	57.000	112.000	−0.216	0.829	0.872
Pain	54.500	109.500	−0.407	0.684	0.722
Stabbing sensation	43.000	98.000	−1.182	0.237	0.283
Electric shock sensation	57.500	112.500	−0.174	0.862	0.872
Tension	27.000	105.000	−2.244	0.025 *	0.030
Stiffness	39.000	117.000	−1.444	0.149	0.180
Crawling sensation	31.000	109.000	−1.986	0.047 *	0.059
Cold sensation	33.000	111.000	−2.270	0.023 *	0.080
Warm sensation	48.000	103.000	−1.078	0.281	0.456
Itching	45.000	123.000	−1.262	0.207	0.346
Tingling	32.000	87.000	−1.889	0.059	0.069
Tickling	55.000	110.000	−0.913	0.361	0.771
Burning sensation	46.500	101.500	−1.034	0.301	0.381
Vibration	54.000	132.000	−1.095	0.273	0.722
Speech difficulty	58.000	136.000	−0.135	0.893	0.923
Salivation	27.500	105.500	−2.350	0.019 *	0.030
Accidental biting	33.000	111.000	−1.841	0.066	0.080

Comparisons between laser and TENS treatment groups before treatment were performed using the Mann–Whitney U test. * *p* < 0.05 was considered statistically significant.

**Table 8:** Comparison of laser and TENS treatment groups after treatment.

Variable	Mann–Whitney U	Wilcoxon W	Z	Asymp. Sig. (2-Tailed)	Exact Sig. (1-Tailed)
Numbness	38.000	116.000	−1.483	0.138	0.159
Pain	56.000	134.000	−0.319	0.749	0.821
Stabbing sensation	57.500	112.500	−0.181	0.856	0.872
Electric shock sensation	55.500	133.500	−0.326	0.744	0.771
Tension	13.500	91.500	−3.161	0.002 *	0.001
Stiffness	10.500	88.500	−3.300	0.001 *	<0.001
Crawling sensation	19.500	97.500	−2.818	0.005 *	0.006
Cold sensation	30.000	108.000	−2.694	0.007 *	0.050
Warm sensation	48.000	103.000	−1.078	0.281	0.456
Itching	45.000	123.000	−1.262	0.207	0.346
Tingling	35.500	113.500	−1.640	0.101	0.107
Tickling	55.000	110.000	−0.913	0.361	0.771
Burning sensation	55.500	110.500	−0.378	0.705	0.771
Vibration	54.000	132.000	−1.095	0.273	0.722
Speech difficulty	27.000	105.000	−2.207	0.027 *	0.030
Salivation	20.500	98.500	−3.032	0.002 *	0.007
Accidental biting	9.000	87.000	−3.425	0.001 *	<0.001

Comparisons between laser and TENS treatment groups after treatment were performed using the Mann–Whitney U test. * *p* < 0.05 was considered statistically significant.

**Table 9:** Comparison of neurosensory symptoms between inferior alveolar nerve and lingual nerve groups in patients treated with laser therapy.

Symptom	IAN Median (Min–Max)	LN Median (Min–Max)	*p*-Value
Numbness	8.0 (7–9)	8.0 (7–9)	1.000
Pain	1.0 (0–5)	1.0 (0–5)	0.589
Biting	3.0 (0–8)	3.0 (0–8)	0.026 *
Electric shock	3.0 (0–7)	3.0 (0–7)	0.485
Tension	0.0 (0–7)	0.0 (0–7)	0.132
Stiffness	5.0 (0–7)	5.0 (0–7)	0.093
Crawling sensation	3.5 (0–8)	3.5 (0–8)	0.015 *
Cold sensation	0.0 (0–8)	0.0 (0–8)	0.699
Warm sensation	0.0 (0–6)	0.0 (0–6)	0.485
Itching	0.0 (0–7)	0.0 (0–7)	0.394
Tingling	6.0 (3–7)	6.0 (3–7)	0.937
Swallowing	0.0 (0–3)	0.0 (0–3)	0.699
Burning	0.0 (0–8)	0.0 (0–8)	0.394
Vibration	0.0 (0–0)	0.0 (0–0)	1.000
Speech difficulty	5.0 (3–7)	5.0 (3–7)	0.041 *
Salivation	0.0 (0–4)	0.0 (0–4)	0.180
Tongue biting	4.5 (0–7)	4.5 (0–7)	0.132
Taste disturbance (pre)	0.0 (0–1)	0.0 (0–1)	0.002 *
Taste disturbance (post)	0.0 (0–1)	0.0 (0–1)	0.002 *

Data are presented as median (minimum–maximum). Comparisons between the inferior alveolar nerve (IAN) and lingual nerve (LN) groups were performed using the Mann–Whitney U test. * *p* < 0.05 was considered statistically significant.

**Table 10:** Changes in neurosensory symptoms before and after laser therapy in patients with lingual nerve injury (N. lingualis).

Symptom	Pre-Treatment Median (Min–Max)	Post-Treatment Median (Min–Max)	*p*-Value
Numbness	8.0 (7–9)	7.0 (0–8)	0.185
Pain	1.5 (0–5)	0.5 (0–2)	0.211
Biting	6.5 (0–8)	3.0 (0–7)	0.012 *
Electric shock	3.0 (0–7)	1.0 (0–5)	0.337
Tension	0.0 (0–5)	0.0 (0–3)	0.181
Stiffness	0.0 (0–5)	0.0 (0–1)	0.182
Crawling sensation	0.0 (0–3)	0.0 (0–3)	0.221
Tingling	6.5 (3–7)	3.0 (1–7)	0.132 *
Speech difficulty	5.5 (0–7)	3.0 (0–5)	0.006 *
Salivation	0.0 (0–4)	0.0 (0–1)	0.317
Taste disturbance	6.0 (5–7)	2.0 (1–3)	0.002 *

Data are presented as median (minimum–maximum). Pre- and post-treatment comparisons were performed using the Wilcoxon signed-rank test. * *p* < 0.05 was considered statistically significant.

**Table 11:** Changes in neurosensory symptoms before and after laser therapy in patients with inferior alveolar nerve injury (N. alveolaris inferior).

Symptom	Pre-Treatment Median (Min–Max)	Post-Treatment Median (Min–Max)	*p*-Value
Numbness	8.0 (7–9)	5.0 (4–7)	0.185
Pain	1.0 (0–4)	0.0 (0–1)	0.211
Biting	1.5 (0–4)	0.0 (0–1)	0.015 *
Electric shock	1.5 (0–4)	0.0 (0–2)	0.337
Tension	5.0 (0–7)	1.0 (0–3)	0.072
Stiffness	6.0 (4–7)	1.5 (0–4)	0.182
Crawling sensation	4.5 (0–7)	1.0 (0–3)	0.021 *
Tingling	6.0 (6–7)	2.0 (1–3)	0.132 *
Speech difficulty	4.0 (3–6)	1.0 (0–2)	0.006 *
Salivation	1.0 (0–4)	0.0 (0–1)	0.317
Taste disturbance	0.0 (0–0)	0.0 (0–0)	–

Data are presented as median (minimum–maximum). Pre- and post-treatment comparisons were performed using the Wilcoxon signed-rank test. * *p* < 0.05 was considered statistically significant.

**Table 12:** Comparison of neurosensory symptoms between lingual nerve and inferior alveolar nerve groups after laser therapy.

Symptom	N. Lingualis Median (Min–Max)	N. Alveolaris Inferior Median (Min–Max)	*p*-Value
Numbness	7.0 (0–8)	5.0 (4–7)	0.240
Pain	0.5 (0–2)	0.0 (0–1)	0.310
Biting	3.0 (0–7)	0.0 (0–1)	0.015 *
Electric shock	1.0 (0–5)	0.0 (0–2)	0.394
Tension	0.0 (0–3)	1.0 (0–3)	0.132
Stiffness	0.0 (0–1)	1.5 (0–4)	0.240
Crawling sensation	0.0 (0–3)	1.0 (0–3)	0.221
Tingling	3.0 (1–7)	2.0 (1–3)	0.180
Speech difficulty	3.0 (0–5)	1.0 (0–2)	0.006 *
Salivation	0.0 (0–1)	0.0 (0–1)	1.000
Taste disturbance	2.0 (1–3)	0.0 (0–0)	0.002 *

Data are presented as median (minimum–maximum). Comparisons between groups were performed using the Mann–Whitney U test. * *p* < 0.05 was considered statistically significant.

**Table 13:** Wilcoxon signed-rank test results comparing healthy and pathological sides before treatment.

Muscle	Task	Z Value	*p* Value
Masseter	Painful—soft bite	−4.558	<0.001 *
Masseter	Painful—maximal force	−4.392	<0.001 *
Masseter	Painful—repetitive movement	−2.687	0.007 *
Masseter	Painless—soft bite	−4.297	<0.001 *
Masseter	Painless—maximal force	−4.467	<0.001 *
Masseter	Painless—repetitive movement	−2.692	0.007 *
Mylohyoid	Painful—soft bite	−4.042	<0.001 *
Mylohyoid	Painful—maximal force	−4.034	<0.001 *
Mylohyoid	Painful—repetitive movement	−2.375	0.018 *
Mylohyoid	Painless—soft bite	−4.209	<0.001 *
Mylohyoid	Painless—maximal force	−4.210	<0.001 *
Mylohyoid	Painless—repetitive movement	−1.841	0.066

* *p* < 0.05 statistically significant.

**Table 14:** Wilcoxon signed-rank test results comparing pre- and post-treatment cutaneous silent period (CSP) durations on the pathological side.

Muscle	Task	Z Value	*p* Value
Masseter	Painful—soft bite	−1.487	0.137
Masseter	Painful—maximal force	−0.984	0.325
Masseter	Painful—repetitive movement	−0.137	0.891
Masseter	Painless—soft bite	−0.829	0.407
Masseter	Painless—maximal force	−0.096	0.924
Masseter	Painless—repetitive movement	−0.333	0.739
Mylohyoid	Painful—soft bite	−2.818	0.005 *
Mylohyoid	Painful—maximal force	−2.617	0.009 *
Mylohyoid	Painful—repetitive movement	−1.511	0.131
Mylohyoid	Painless—soft bite	−1.254	0.210
Mylohyoid	Painless—maximal force	−2.406	0.016 *
Mylohyoid	Painless—repetitive movement	−1.890	0.059

Wilcoxon signed-rank test was used to compare pre- and post-treatment cutaneous silent period (CSP) durations on the pathological side. * *p* < 0.05 was considered statistically significant.

## Data Availability

Data are available on request due to privacy/ethical reasons.
